# A Growing Evil Which Should Be Remedied

**Published:** 1892-07

**Authors:** E. A. Cobleigh

**Affiliations:** Chattanooga, Tenn.


CHATTANOOGA, TENN.

EDITOR SOUTHERN MEDICAL RECORD:

*Dear Sir*—In your last issue I read an editorial on "A Growing Evil which should be Remedied," and your suggestions of the remedy, with the natural interest which any physician would feel who has practiced a few years in a place where prescription writing is the rule, because such practitioner will without doubt have been a sufferer to some extent from the very evil you mention. Yet it is the same old story which has been going the round of the medical and drug periodicals, and has been accompanied with much differing and even acrimonious dispute, for years and years. And still the thing continues and grinds along, just as before.

Now the druggist is a great convenience to the busy doctor without doubt, and he relieves him likewise of the necessity of carrying a big cash investment in drugs for office use, and he is not a man we wish to quarrel with. He is our friend so far as mutual trade interests go. But when he adopts the iron clad rule not to refill prescriptions unless directly on order of the prescriber, his business is quite likely to largely fly away to his competitor, because usage at least is along the other course, without necessitating the discussion of its right or error in this article. So we accept the fact as it stands, and look about for relief from the evil, with its attendant injustice to the physician, and liability of injuring the patient or those friends to whom he ignorantly but kindly loans the formula.

You suggest printing the order "not to be refilled," etc., on the blank. But it is a fact that in spite of this instruction, it is more often ignored than observed, like many printed injunctions which we daily become familiar with violating, mainly from the American habit of ideal liberty ,which implies resentment of its attempted abridgment. And further, it is a well known fact that the courts have again and again decided (in spite of all arguments by the medical and drug professions that they are severally the owners of this little piece of paper after it is written), that the prescription is the indisputable property of the purchaser, the patient, who has paid his fee for the same. Wherefore the only legal way of protection is by furnishing the medicine in some manner which does not commit the formula to paper. This would mean by verbal direction to the druggist, or by 'phone message, or by speaking tube, etc., all being more or less impracticable. Wherefore it long ago occurred to the writer that in diplomacy, without useless contention over facts as they exist, lay the remedy, and he applied his reasoning to practice, with perfect satisfaction.

This was accomplished by stocking his office with a small line of drugs, mainly pellets, tablet triturates, and things of small bulk (and it might also be said of small cost and equally small medicinal utility) not for the purpose of medicating the patient as much as for protection of the practitioner. And this done, there is little trouble in making the patient return to your office if his case fails to progress satisfactorily, or he needs repetition of like remedies in the course of future months. How? When the patient receives a prescription from the medical man, he likewise receives a small package of pellets, sugar or milk tablets will answer as well as anything else, but it is best to have a variety of differing forms and colors and sizes of these things, so as not always to use something which the recipient will recognize as given before. Full directions regarding the medicine which is to be obtained at the drug store are then given and equally minute directions follow regarding the use of the remedy handed out by the doctor. The impression is thus made on the patient's mind that both are equally essential to his treatment, and he can but return to his adviser when the little box or paper received from him is exhausted. See? It is the easiest thing in the world, entails scarcely any loss of time, very little outlay, and compasses the practical solution of a problem about which much bitterness has resulted between doctor and druggist and patient, with absolutely not the least ruffling of anybody's feelings. Let those who suffer in this way from the abuse of prescriptions by patrons, just try this method, and they will be surprised how smoothly and how reliably it works, thus demonstrating how much better it is to win by strategy, than to attempt the forced acceptance of a reform which has bitter antagonisms. Very truly yours,

E. A. COBLEIGH, M. D.

---

It is with pleasure we direct attention to the advertisement of the Bovinine Co., formerly the J. P. Bush Co. Their raw food extract stands alone and is undisputedly the most concentrated and nutritious food extant. After a career of fourteen years, it is now extensively prescribed by the medical profession, because of its material excellence, clinical efficiency and great economy.

